# Supplementary material for: The interactome of intact mitochondria by cross-linking mass spectrometry provides evidence for coexisting respiratory supercomplexes
Source: Mol Cell Proteomics. 2017 Dec 8;17(2):216–32. doi: 10.1074/mcp.RA117.000470 (PMC5795388; doi:10.1074/mcp.RA117.000470)
Supplement: Supplemental Data [file supp_RA117.000470_133922_0_supp_23978_fzffwf.zip › spectra_annotation/mito_spectra_annotation/73-1-7-1-8-1.pdf]

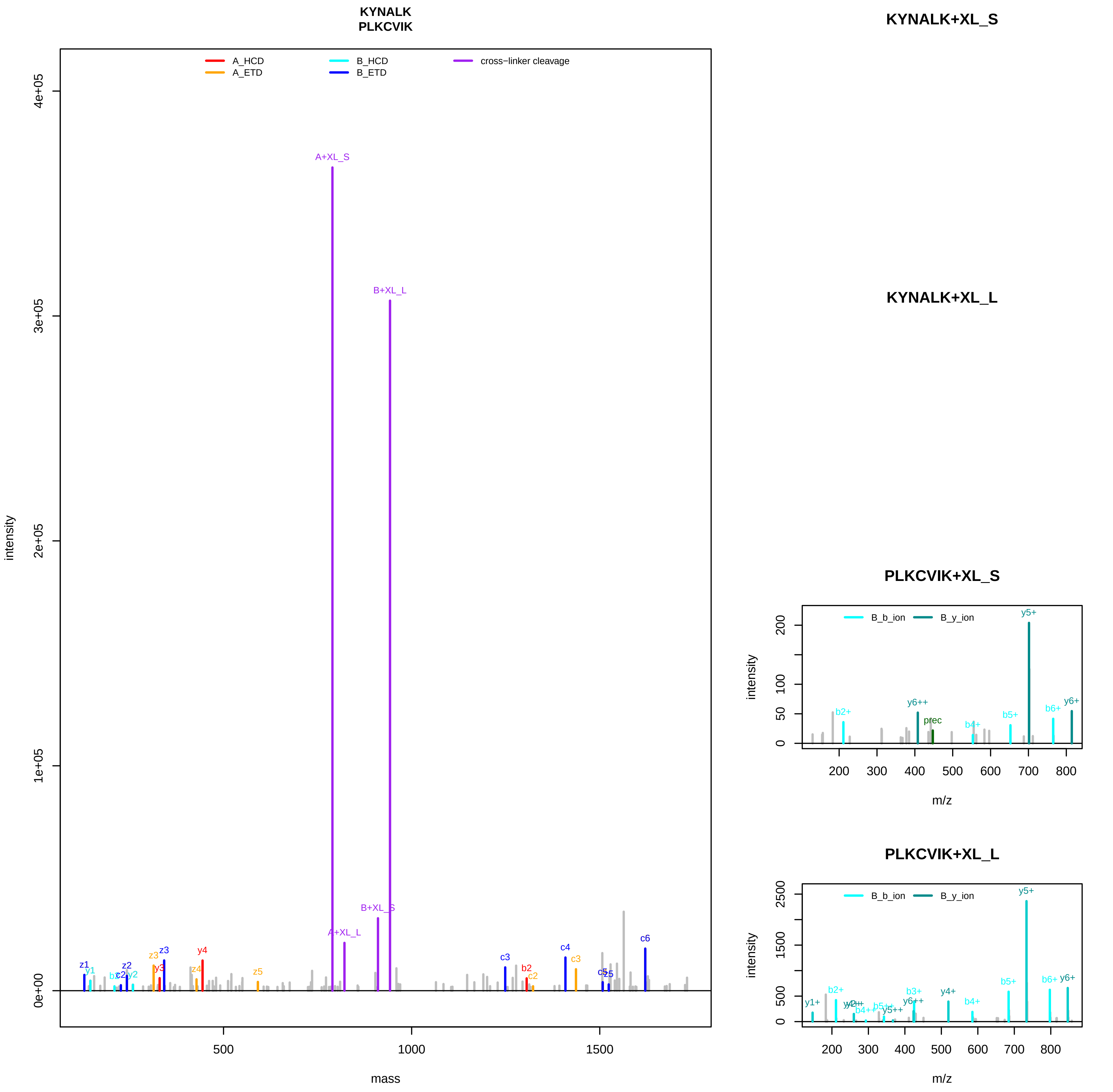
KYNALK
PLKCVIK
A_HCD
A_ETD
B_HCD
B_ETD
cross−linker cleavage
A+XL_S
B+XL_L
B+XL_S
A+XL_L
z1
y1
b2
c2
z2
y2
z3
y3
y4
z4
z5
c3
b2
c2
c4
c3
c5
z5
c6
intensity
mass
0e+00
1e+05
2e+05
3e+05
4e+05
500
1000
1500
KYNALK+XL_S
KYNALK+XL_L
PLKCVIK+XL_S
B_b_ion
B_y_ion
y5+
y6++
b2+
prec
b4+
b5+
b6+
y6+
0
50
100
200
300
400
500
600
700
800
m/z
PLKCVIK+XL_L
y1+
y2+
y2++
b4++
b5++
y5++
b3+
y6++
y4+
2500
1500
500
